# Methylene Blue in a High-Performance Hydrogen-Organic Rechargeable Fuel Cell

**DOI:** 10.1021/acsaem.3c02515

**Published:** 2024-03-06

**Authors:** Christopher G. Cannon, Peter A. A. Klusener, Luke F. Petit, Toby Wong, Anqi Wang, Qilei Song, Nigel P. Brandon, Anthony R. J. Kucernak

**Affiliations:** † Department of Chemistry, Imperial College London MSRH, White City, London W12 0BZ, U.K.; ‡ Shell Global Solutions International B.V., Energy Transition Campus Amsterdam, Grasweg 31, 1031 HW Amsterdam, The Netherlands; § Department of Chemical Engineering, Imperial College London, South Kensington, London SW7 2AZ, U.K.; ∥ Department of Earth Science and Engineering, Imperial College London, South Kensington, London SW7 2AZ, U.K.

**Keywords:** energy storage, methylene blue, hydrogen, fuel cell, flow battery, PBI, electrochemical, stable

## Abstract

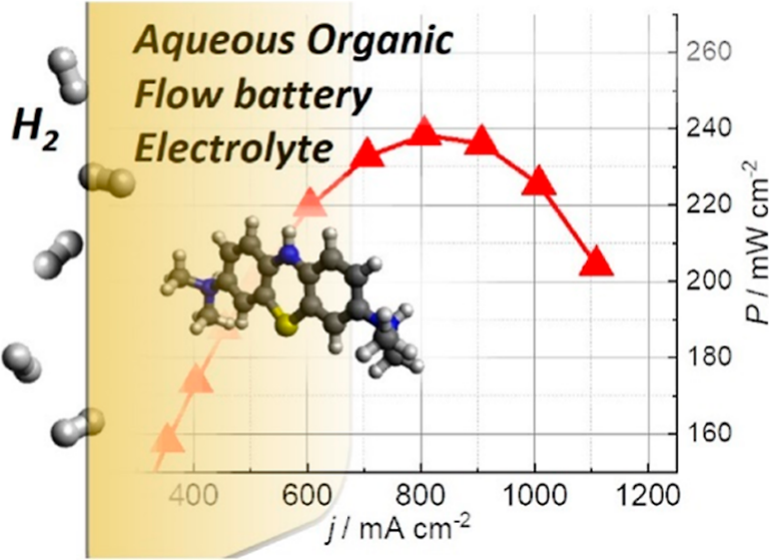

A hydrogen-organic hybrid flow battery (FB) has been developed using methylene blue (MB) in an aqueous acid electrolyte with a theoretical positive electrolyte energy storage capacity of 65.4 A h L^–1^. MB paired with the versatile H_2_/H^+^ redox couple at the negative electrode forms the H_2_–MB rechargeable fuel cell, with no loss in capacity (5 sig. figures) over 30 100% discharge cycles of galvanostatic cycling at 50 mA cm^–2^, which shows excellent stability. A peak power density of 238 mW cm^–2^ has also been demonstrated by utilizing 1.0 M MB electrolyte. This represents a type of scalable electrochemical energy storage system with favorable properties in terms of material cost, stability, crossover management, and energy and power density, overcoming many typical limitations of organic-based redox FBs.

## 1 Introduction

For all types of energy infrastructure, the cost and security of energy supplies are critical economic issues worldwide. As the means of producing grid electricity evolves, renewable energy represents a growing fraction.^1^ However, solar and wind energy are intermittent resources, and only a few energy storage systems are adaptable to storing grid-scale quantities of energy in an economically viable way. The flow battery (FB) has been considered a scalable design of electrochemical energy storage (EES).^2^

The traditional FB pumps liquids containing redox-active species through liquid flow channels in the cells adjacent to porous electrodes. These electrodes are typically made of graphitic carbon felts or cloths. The positive and negative sides are separated by an ion-exchange membrane (IEM) or porous separator in a zero-gap arrangement. This allows the energy storage capacity (*C*) to scale with the amount of external electrolyte, independent of the cell stack size. The all-vanadium FB (VFB) is a commonly referenced example, as it is arguably the most mature FB chemistry.^3^ The VFB stores and releases energy using the conversion between dissolved V^II^/V^III^ salts on the negative side and V^IV^/V^V^ salts on the positive side, both in aqueous sulfuric acid solutions.

A key challenge is to lower the cost of the electrolyte solutions. There are many chemistries other than vanadium that have been demonstrated, with electrolytes spanning the pH range, notably quinones, TEMPO, and ferrocene.^4^ Recently, *organic* energy storage molecules have been considered to reduce the electrolyte cost and provide better price stability, often benchmarked by the price of vanadium. Organic electrolytes could therefore make FB technology considerably more competitive in the grid-scale EES market. Over the previous 2–3 decades, the research output into alternative FB chemistries has proliferated, one class of which is all-organic redox couples for aqueous electrolyte systems.^4,5^ One critical issue faced with many organic FBs is long-term capacity retention. Active material degradation leads to capacity loss, and considering that prospective EES devices would be required to operate for multiple decades, many FB chemistries that suffer from degradation are unlikely to be carried forward. Another cause of long-term degradation is the crossover of the active species to the opposite side of the cell. This can be improved with more selective IEMs or mitigated by using a gas–liquid rechargeable fuel cell (RFC) arrangement, whereby one of the liquid electrolytes is replaced with a gas.

Hydrogen is an excellent energy storage medium, with fast and reversible kinetics to form protons when oxidized in an acidic environment, using platinum group metal catalysts.^6^ The round-trip efficiency of producing hydrogen in a water electrolyzer and consuming it in a H_2_–O_2_ fuel cell is typically quite low (<50%). Replacing O_2_ with a more reversible redox couple has inspired the development of alternative H_2_–*X* systems, where *X* is a protic FB electrolyte. Methylene blue (MB) is a ubiquitous phenothiazine dye molecule and the first synthetic drug.^7^ It was widely used for its antiseptic properties and continues to be used today in the treatment methemoglobinemia in humans.^8^ MB is produced at a large scale and is available at 3–9 $ kg^–1^ (i.e., 0.03–0.1 $ kW h^–1^ for the system described here) when purchased at the multi-kg scale.^9^ In this work, we paired MB with hydrogen to demonstrate a fully aqueous RFC, using various proton-exchange membranes (PEMs) to realize stable cycling performance. These include *Nafion 212*, a 4,4′-diamine-3,3′-dimethyl-biphenyl Tröer’s base (DMBP-TB) membrane, and a commercially available polybenzimidazole (PBI) fuel cell membrane. At a 0.1 M MB concentration, the H_2_–MB RFC utilizing a PBI membrane could be cycled for more than 3 days at 50 mA cm^–2^ with no capacity loss and >76% round-trip efficiency. Furthermore, the power density at 0.1 M could exceed 200 mW cm^–2^ at 100% state of charge (SOC). The specific capacity of the positive MB electrolyte can reach 65.40 A h L^–1^, which is equivalent to 2.4 mol e^–^ L^–1^. If coupled with a H_2_ storage tank on the negative side to form a closed system, this is equivalent to 13.67 bar compressed H_2_ (Supporting Information, Section S2).^10^ In this case, the energy density of the H_2_–MB RFC, considering both the positive and negative tank volumes, would be 14.81 W h L^–1^; or 54.65 W h L^–1^ with H_2_ storage at 100 bar.

## 2 Experimental Section

### 2.1 Chemicals and Material Characterization

Methylene blue chloride (Thermo Fisher Scientific) was used as received. Electrolyte solutions were prepared from 95% H_2_SO_4_ (VWR) and 18.2 MΩ·cm ultrapure water from a Sartorius purification system. Rotating disk electrode (RDE) measurements were performed using a polished glassy-carbon disk of 5 mm diameter as the working electrode and an RDE rotor (Pine Instruments), with a saturated calomel reference electrode and a graphite rod counter electrode in a 3-compartment cell. Voltammograms were recorded on an Autolab PGSTAT302N at a scan rate of 10 mV s^–1^ using freshly prepared 1 mol dm^–3^ H_2_SO_4_ solutions containing 1 mmol dm^–3^ MB analyte, which were purged with argon (Air Products, BIP Plus N6.6) prior to each measurement. The kinetic rate constant and diffusion coefficient were calculated as described in the Supporting Information in Section S3. Cyclic voltammetry measurements used a reversible hydrogen electrode (RHE). For the solubility determination, 10 mmol samples of MB were stirred for at least 24 h with H_2_SO_4_ solutions of various concentrations at room temperature and syringe-filtered through a 0.2 μm PTFE membrane (Puradisc 25 TF, Whatman) to remove undissolved solids from the electrolyte. The filtrate was diluted using ultrapure water to within the range of calibration where the Beer–Lambert law was applicable (Supporting Information, Section S5). The synthesis of the DMBP-TB polymer followed previous reports.^11^*Nafion* 212 was pretreated by soaking the membrane in 5% H_2_O_2_ for 1 h at 80 °C. This was repeated with ultrapure water, then 1 M H_2_SO_4_. DMBP-TB membranes were pretreated in 1 M H_2_SO_4_ overnight before use. Two PBI membrane types (Celtec, BASF) were used—one which was saturated with phosphoric acid, and one without any phosphoric acid in it but which was hydrated with pure water (phosphoric acid free). Both membranes were used as received.

### 2.2 Membrane Electrode Assembly and RFC Testing

The positive-side electrode used a layer of 4.6 mm carbon felt (*SIGRACELL*), which was oxygen-plasma-treated (*Diener* “*nano*” low-pressure plasma system) before assembling the membrane electrode assembly (MEA) to remove surface impurities from the electrode felt. For the H_2_ side, a 190 μm, 0.4 mg_Pt_ cm^–2^ electrode (*ELE0201, Johnson Matthey Fuel Cells*) was used as received. The electrodes, membranes, and gaskets (*Tygaflor*) were compressed with 4.0 N m applied torque in a 5 cm^2^ flow cell fixture (*Scribner Associates*) to produce ∼20% electrode compression relative to the original electrode thicknesses. Full cell experiments were recorded using a *Scribner 857 RFB test station*. The MEA was prepared as described above, specifically using a phosphoric acid free *Celtec* PBI membrane. Solutions were prepared by dissolving the MB in 6 M H_2_SO_4_ (up to 1.0 M) and 7 M H_2_SO_4_ (1.2 M). Galvanostatic charge–discharge cycles were performed using a 5 cm^2^ cell at ±50 mA cm^–2^ to the maximum possible depth of discharge within cell voltage limits of 0.9 and 0.3 V. During polarization, the discharge current was increased stepwise. All measurements were conducted at room temperature. Hydrogen gas was produced from an electrolyzer (*60H-FUEL Hydrogen Generator*, *Parker*) and flowed at a rate of 100 mL min^–1^ (1 bar), set using a H_2_ mass-flow controller (*El-Flow Select, Bronkhorst*). The H_2_ humidity of the RFC hydrogen inlet line was measured at room temperature by using a dew-point transmitter (*Optidew dew-point transmitter, Michell Instruments*). During polarization experiments, the H_2_ relative humidity was set at 98–100% by flowing through a humidification column (*Perma Pure MH-110-12S-2*) before the cell inlet. The positive electrolyte was flowed at a constant rate of 50 mL min^–1^ without any protection from air.

## 3 Results and Discussion

**Scheme 1:**
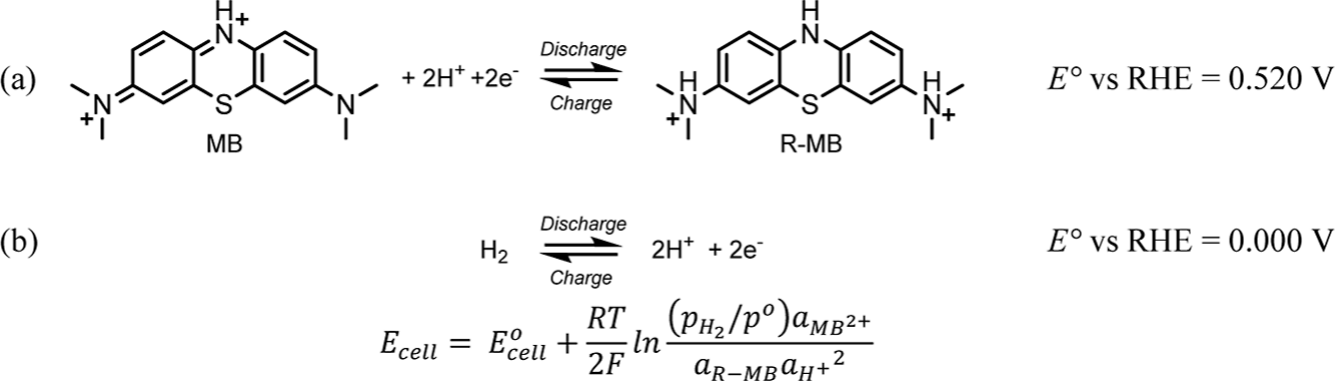
(a) Positive-Side and (b) Negative-Side H_2_–MB Cell Reactions

The preliminary electrochemical characterization of MB in sulfuric acid showed that the reaction was sufficiently reversible to warrant its application in the RFC (Section S1). The two-electron transfer occurs in a single reversible wave, and the half-wave potential at 0.520 V vs RHE in 1 M H_2_SO_4_ corresponds to the H_2_-MB cell voltage. Like many organic molecules, it exhibited a two-electron energy storage capacity per molecule, and Zhang et al. recently showed that at low pH, the redox mechanism is a proton-coupled 2H^+^/2e^–^ transfer.^12^ This implies that in strong acid, the positive electrolyte cycles between the MB and reduced (R-MB) structures shown in Scheme 1a. Di-protonation of R-MB buffers the proton efflux and influx during cell charging and discharging, respectively, with the hydrogen redox couple (Scheme 1b). Assuming the ionic properties of sulfate and bisulfate ions are the same in both states of charge, the positive electrolyte pH would remain reasonably constant.

**Figure 1:**
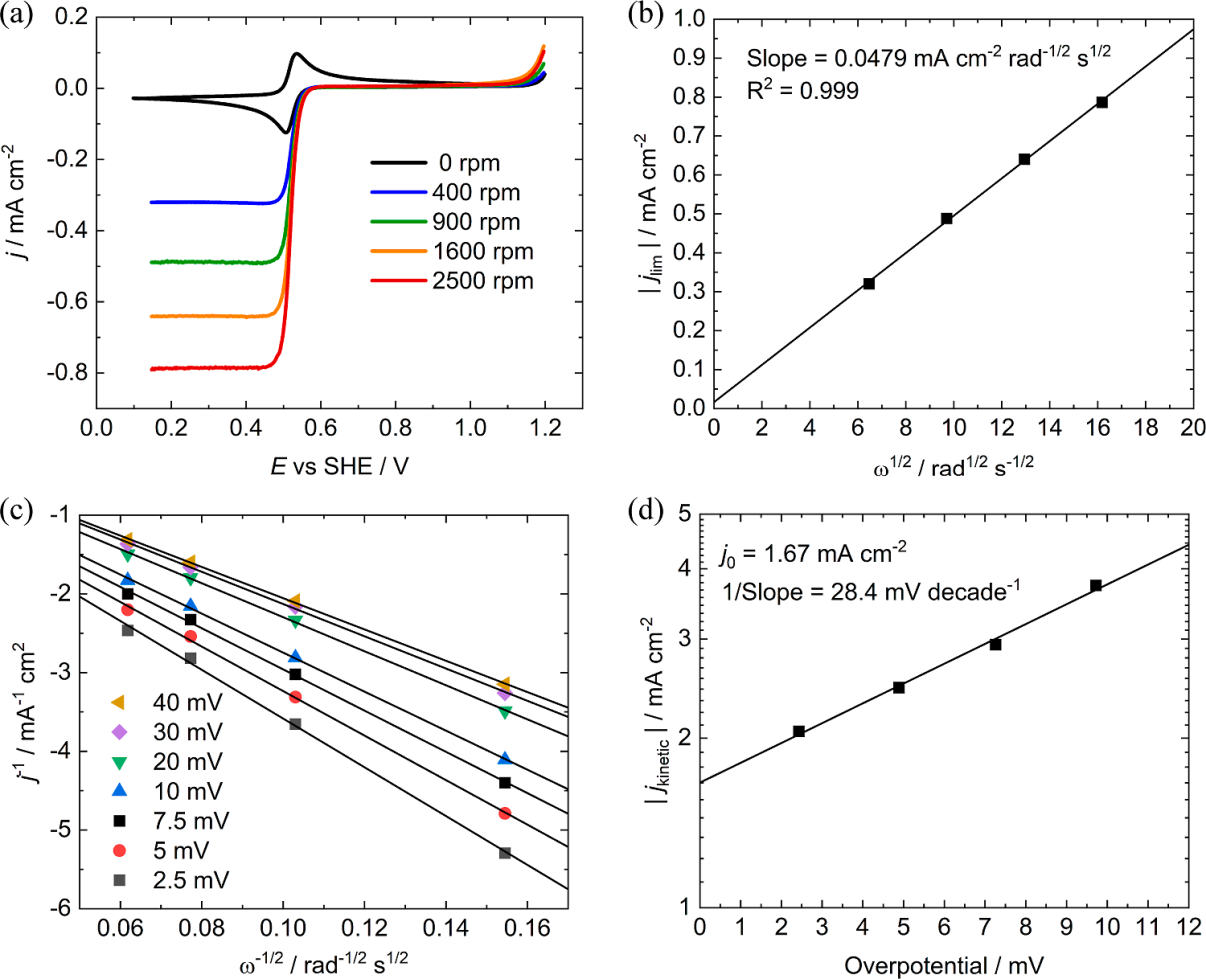
Koutecký–Levich analysis of MB voltammetry—(a) *iR*-free and background-corrected RDE voltammetry profiles with 10 mV s^–1^ scan rate of 1 mM MB/argon-sat. 1 M H_2_SO_4_ solutions as a function of glassy-carbon disk rotation frequency, (b) Levich plot of the limiting current vs the square root of the rotation rate between 400 and 2500 rpm, (c) Koutecký–Levich plot for the inverse of the RDE current at different overpotentials between 2.5 and 40 mV, and (d) absolute kinetic current density vs overpotential used to estimate the exchange current density.

The kinetic rate constant *k*_e_ for electrochemical MB reduction on glassy carbon (Figure 1) was found to be 8.65 × 10^–3^ cm s^–1^ and the diffusion coefficient *D* is 1.51 × 10^–6^ cm^2^ s^–1^ (see Supporting Information Section S3 for details of calculation). The kinetic rate constant of the electron-transfer reaction is therefore high, and the diffusion coefficient is of similar magnitude to other reported organic and metallic FB electrolytes.^3,13−16^

**Figure 2:**
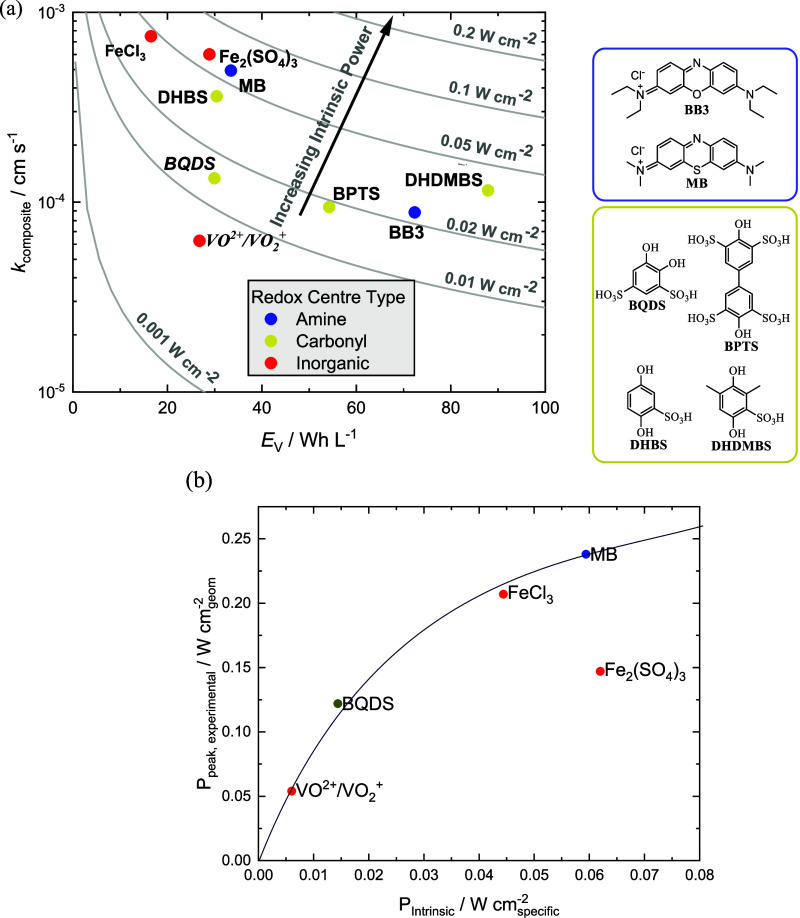
(a) Plot of the theoretical “intrinsic power” (*P*_intrinsic_) for MB and other positive electrolyte FB redox couples. *P*_intrinsic_ is a measure of the theoretical performance of a redox couple paired with the (much faster) hydrogen reaction. BB3^22^ = basic blue 3, MB = methylene blue, BQDS^23^ = 1,2-dihydrobenzoquinone-3,5-disulfonic acid, BPTS^24^ = 4,4′-biphenol-3,3′,5,5′-tetrasulfonic acid, DHBS^23,25^ = 1,4-dihydrobenzoquinone-3-sulfonic acid, DHDMBS^23^ = 3,6-dihydroxy-2,4-dimethylbenzenesulfonic acid; (b) correlation of the literature values of peak power of H_2_–X FBs^17−19^ with different chemistries to the “intrinsic power” (*P*_intrinsic_), of each redox system.

We can use the parameters *k* and *D*, as well as the energy storage capacity of an aqueous FB electrolyte, to compare MB to other redox couples. This can be done in terms of the notional *iR*-free theoretical power density in a model RFC (Supporting Information, Section S3).^5^ The *y*-axis in Figure 2 uses a composite rate constant *k*_composite_, which considers the intrinsic rate of the redox-active species to diffuse in solution and the kinetic rate of overcoming the activation energy barrier to switch between oxidation states. The *x*-axis is the volumetric energy density of the positive electrolyte in a H_2_–*X* RFC configuration. All values are normalized to the surface-area specific power of a Fe^2+^/Fe^3+^ model system (Table S2, Supporting Information).^5^ This is a ranking of the properties of *energy density*, *electron-transfer kinetics*, and *diffusivity* into the *intrinsic power* (*P*_intrinsic_). The *P*_intrinsic_ is a notional term which is characterized as being the power at the electrode surface (i.e., at unit roughness) under constant flow conditions and at a constant overpotential. Hence, this power normalizes its value to the properties of the molecule rather than the FB electrolyte solution and cell conditions.^5^ We suggest that *P*_intrinsic_ is a good predictor of the performance of a redox couple within an operating RFC, when that redox couple is the limiting factor to performance.

There are many different cell chemistries that can already be classified as H_2_–*X* RFCs. These include, but are not limited to, vanadium, 1,2-dihydrobenzoquinone-3,5-disulfonic acid (BQDS), iron, cerium, and bromine.^17−21^ Of the intrinsic power values plotted in Figure 2 using data gathered from the literature, the most comparable to MB are from other H_2_–*X* systems. The faster redox kinetics and higher volumetric energy density of the MB electrolyte presented here give a higher *P*_intrinsic_ compared to VO^2+^/VO_2_^+^, and the H_2_–MB system indeed outperforms early iterations of the H_2_–V RFC in terms of the peak power density achieved at 100% SOC.^17^ Iron RFCs typically use chloride- and sulfate-based counterion/electrolyte formulations. Iron sulfate (1.4 M Fe) and iron chloride (0.9 M Fe) in a comparable H_2_–Fe RFC system have achieved slightly lower peak power densities (147 and 207 mW cm^–2^, respectively) than the H_2_–MB system in this work.^19^

Organic redox couples often undergo a two-electron-per-molecule redox switch. In Figure 2a, we include the theoretical points of the *P*_intrinsic_ of a number of organic molecules, only one of which (BDQS) has been tried in a H_2_–X RFC. Basic blue 3 (BB3) is one example of a dye molecule with structural features similar to those of MB, but the two-electron redox reversibility is much more sluggish. The biphenol molecule 4,4′-biphenol-3,3′,5,5′-tetrasulfonic acid (BPTS) shows a similar value of *P*_intrinsic_ but achieves this with slower kinetics and a higher energy density.

Examples of quinones included here are BDQS, 1,4-dihydrobenzoquinone-3-sulfonic acid (DHBS), and 3,6-dihydroxy-2,4-dimethylbenzenesulfonic acid (DHDMBS). Quinone derivatives are prevalent candidates for low-pH organic FB electrolytes.^5^ The fast *k*_composite_ and higher energy density of the MB electrolyte in this work compared to 0.65 M BQDS are reflected both by a higher *P*_intrinsic_ value and experimental peak power (238 mW cm^–2^ for H_2_–MB, compared to 122 mW cm^–2^ for H_2_–BQDS, at room temperature and 100% SOC).^18^ This validates the assertion that *P*_intrinsic_ is a good predictor of the performance of a redox couple within a H_2_–X RFC. The energy storage capacity and electrochemical properties of MB are therefore relatively promising and translate into real-world performance.

Figure 2b shows the *intrinsic power* of the redox couple where the peak power data come from a number of studies using these redox molecules.^17−19^ The peak power of a H_2_–X FB is well-correlated to *P*_intrinsic_, showing the same ordering apart from the case of Fe_2_(SO_4_)_3_ (discussed below). Conversion from *P*_intrinsic_ to peak power is difficult as it depends on a large number of factors including the roughness factor of the electrode, electrolyte flow rate, flow field geometry, etc. Nonetheless, *P*_intrinsic_ is useful in assessing the ranking of couples and sets an “upper bound” on performance for a well-optimized FB. Hence, the *P*_intrinsic_ is a good possible indicator for the performance of a H_2_–X FB. Although Fe_2_(SO_4_)_3_ would appear to be a good possible redox species (having a *P*_intrinsic_ similar to that of MB), its peak power is much less than might be expected. This might be due to a number of reasons such as the high viscosity of the Fe_2_(SO_4_)_3_ electrolyte^19^ reducing mass transport rates; extensive ion pairing of this species in solution [forming FeSO_4_^+^, Fe(SO_4_)_2_^–^, and FeSO_4_ solution species^26^ leading to reduced electron-transfer rates or a shift in the equilibrium potential; or lack of optimization of the system for this specific electrolyte.

**Figure 3:**
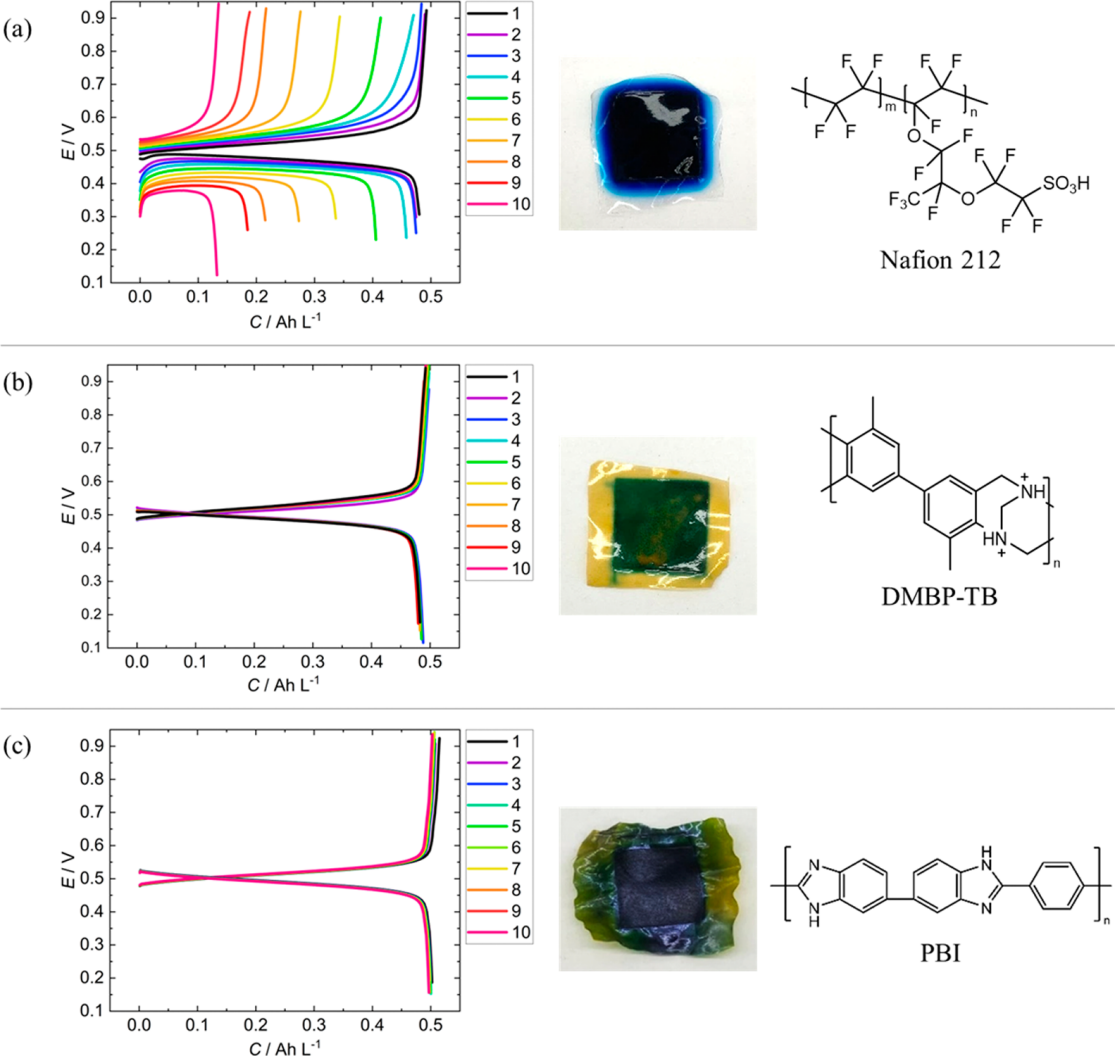
10 mM (50 mL) galvanostatic cycling tests at 20 mA cm^–1^; membrane appearance after 10 cycles; and the polymer membrane structure for an IEM of (a) *Nafion* 212; (b) DMBP-TB; and (c) PBI.

Maintaining good transport of H^+^ above all other ions through the membrane is critical in the H_2_–*X* RFC arrangement. The transport of H^+^ from the positive electrolyte provides a reactant for the hydrogen evolution reaction, and the transference number of H^+^ should be 1. In order to determine the stability of polymer electrolytes in the redox medium, the performances of three different membrane materials (*Nafion*, DMBP-TB, and PBI) were tested in a 10 mM H_2_–MB RFC, which was cycled through ten charge–discharge cycles (Figure 3). *Nafion* is the brand name of Chemour’s synthetic polymer with the structure shown in Figure 3a, and *Nafion* IEMs have been used in low-temperature PEM fuel cells, as well as the conventional choice of membranes in the H_2_–*X* RFC systems developed thus far.^17,27,28^ DMBP-TB is an intrinsically microporous polymer which has been applied in FBs, fuel cells, and nanofiltration applications.^29−31^ The manufacture of such hydrocarbon membranes could be significantly cheaper and arguably safer than *Nafion*, which is a per- and polyfluoroalkyl substance. PBI is a thermoplastic polymer that forms an IEM that is resistant to high temperature and an oxidizing environment. Applications include high-temperature PEM fuel cells, which typically operate in the range of 120–180 °C. Like DMBP-TB, the PBI undergoes protonation in acidic conditions, although the majority of proton conduction occurs through the free acid which is incorporated into the porous structure. The DMBP-TB and PBI membranes resisted any unfavorable interaction with the organic MB cations in the RFC and maintained proton conductivity for the duration of the 10 cycles. Hydrocarbon-based membranes in conjunction with inexpensive organic redox electrolytes in systems such as this could greatly reduce the cost of conventional FB materials and components.

After cycling, the appearance of the membranes all showed staining by the electrolyte, which is unsurprising considering the very high molar absorption coefficient of the dye. In the tests with DMBP-TB and PBI, there was no significant capacity loss over ten charge/discharge cycles. In the case of the *Nafion*-containing MEA test, up to 27 μmol cm^–2^ may have been lost to impregnation of the MB/R-MB into the membrane, as the final concentration of the electrolyte was determined to be only 7.3 mM. The interaction with *Nafion* could be rationalized by the strong affinity between the highly acidic tethered sulfate groups of the IEM and the positively charged organic molecule, an interaction which has previously been used to develop analytical sensors.^32−34^

The charging capacity of the *Nafion* IEM cell diminishes with each cycle, as the cell prematurely reaches the 0.9 V cutoff each cycle (Figure 3a). The value of the high-frequency resistance (HFR) measured at 7.5 kHz corresponds to a large proportion of the total overpotential (Section S4). The HFR sharply increases in each charging process, although decreases during discharging, when the direction of ion transport is reversed. This could indicate that H^+^ transport (expected to have the largest transport number) to the negative electrode is being impeded, causing rapid H_2_–MB cell failure. MB molecules in the widest dimension are 1.43 nm on average.^35^ This is on the same order as the hydrophilic channels in *Nafion*, and fouling from a “plugging” effect reported elsewhere is also possible.^36^ DMBP-TB shows good resistance to the acidic organic electrolyte but could not maintain long-term physical integrity in the liquid–gas arrangement. The phosphoric acid (PA)-doped BASF *Celtec* membrane (PBI) not only shows comparable performance to DMBP-TB but also showed better structural physical integrity after prolonged use.

Sulfuric acid is an inorganic acid that was used as the supporting electrolyte. The solubility of MB in sulfuric acid solutions of various ionic strengths was investigated (Supporting Information, Section S6, Table S3, and Figure S3b). Between 1 and 7 M, more concentrated acid produced MB solutions of higher concentration, although such dyes are known to self-aggregate over time.^37^ If an insufficient amount was added to a certain amount of MB powder, the solid and the sulfuric acid solution would mix to become a wetted paste-like solid, often with a gold-colored luster (Supporting Information, Figure S4), from which a solution and residue phase were indistinguishable. The balance of solvent added to form a liquid phase without totally dissolving the MB was found by using a molar ratio of 1.8:1 to 2.0:1 H_2_SO_4_:MB, depending on the sample. Zhang et al. were able to achieve a solubility in excess of 2.0 M in acetic acid and approximately 1.8 M in a mixture of sulfuric acid and acetic acid.^38^ At approximately 4 M, the ionic conductivity of sulfuric acid solutions peaks at 0.82 S cm^–1^ at 25 °C. This is high compared to acetic acid solutions, which peaks at under at 0.019 S cm^–1^.^39^ The resistivity of FB electrolytes contributes to the series resistance of the cell; therefore, avoiding the use of poorly conductive solvents may be preferable. We found that the concentration of MB in sulfuric acid solutions can reach at least 1.22 mol dm^–3^, with a molality of just over 0.9 mol kg^–1^. This is equivalent to a single-tank capacity of 65.40 A h L^–1^. Considering both the MB–H_2_ cell potential and the capacity, the theoretical positive electrolyte energy density of the positive electrolyte solution is 34.0 W h L^–1^, which is greater than for 1.0 M V^IV/V^ in a H_2_–V system.^40^

**Figure 4:**
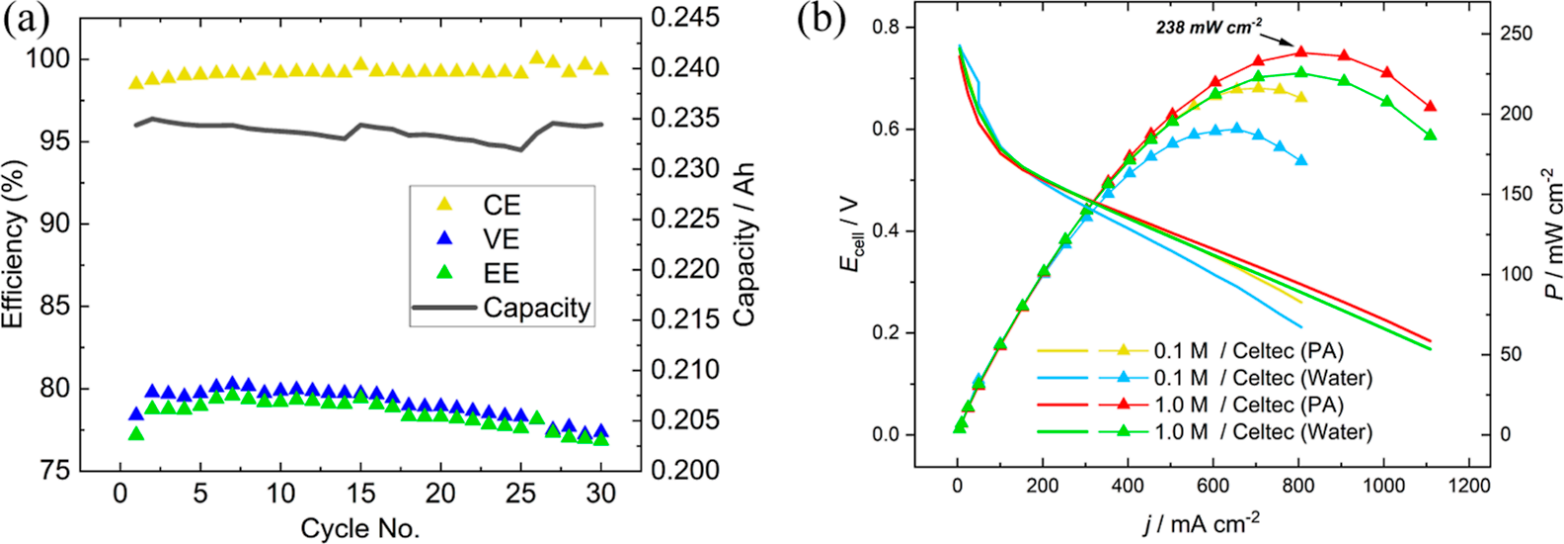
(a) Figures of merit for 0.1 M MB RFC galvanostatic cycling at 50 mA cm^–2^, (b) polarization curves of 0.1 M and 1.0 M MB. Solutions prepared by addition using 6 M H_2_SO_4_ as the supporting electrolyte. PA: Phosphoric acid.

An MEA containing a water-doped *Celtec* PBI membrane was assembled, and a 0.1 M H_2_–MB cell was cycled at 50 mA cm^–2^ through 100% capacity usage of the electrolyte. The round-trip energy efficiency remained over 76% and there was no observed capacity loss in this time period (Figure 4a). Figure 4b shows the discharge power density of a fully charged electrolyte containing 0.1 and 1.0 M MB.

During the long-term cycling experiment, the electrolyte liquid collected in the outlet of the H_2_ gas stream was physically returned to the liquid side. This correlated to the restoration of the original cell capacity observed in cycles 15 and 25. The ability to return crossover redox species to their original tank is a benefit of the liquid–gas approach. The slight decrease in the voltage (and energy) efficiency during long-term cycling is predominantly due to a gradual increase in the overpotential during charging (Figure S5). In the H_2_–MB cell, the overpotential during charging was significantly greater when the RFC was tested at a 1.0 M concentration. This is thought to be characteristic of the liquid–gas arrangement and requires further investigation, as this was not observed for a symmetric 1.2 M MB–MB cell cycled at 200 mA cm^–2^ (Supporting Information, Figure S6), in which the charging profile became only slightly higher over time.

## 4 Conclusions

This work has expanded upon the workable chemistries of the H_2_–*X* RFC and on the emerging field of organic FBs by identifying MB as a stable and reversible redox couple that can show a high working power density, energy density, and stability using a fully aqueous supporting electrolyte. Although *Nafion* was not found to be a suitable electrolyte for this redox couple, two other alternatives have been tested with satisfactory results, suggesting that other polymer electrolytes may be suitable. Although the energy density of the system proposed here is much lower than those of lithium-ion batteries, it possesses fundamental advantages in terms of cost, safety, and scalability. The simplicity of the hydrogen-organic RFC may consequently be further propitious when considering the manufacture and end-of-life processes. Future work in this area also must address the challenge of exploring organic redox couples with more positive redox potentials in order to increase the efficiency and overall commercial appeal of hydrogen-organic RFCs.

## Supporting Information Available

Cyclic voltammetry; theoretical cell voltage and energy density; theoretical power density calculated for various redox couples; *Nafion* cell cycling HFR analysis; effect of sulfuric acid concentration on energy density; photographs of sulfuric acid/MB composite; and performance of the MB symmetrical cell (PDF)
