# Supplementary material for: Methylene Blue in a High-Performance Hydrogen-Organic Rechargeable Fuel Cell
Source: ACS Appl Energy Mater. 2024 Mar 6;7(6):2080–7. doi: 10.1021/acsaem.3c02515 (PMC10966650; doi:10.1021/acsaem.3c02515)
Supplement: Supplementary file 1 — ae3c02515_si_001.pdf [file ae3c02515_si_001.pdf]

Supporting Information

Methylene Blue in a High-Performance Hydrogen-Organic Rechargeable Fuel Cell

*Christopher G. Cannon[a], Peter A. A. Klusener[b], Luke F. Petit[c] , Toby Wong[c], Anqi Wang[c], Qilei Song[c], Nigel P. Brandon[d], and Anthony R. J. Kucernak[a, *]*

[a] Department of Chemistry, Imperial College London

MSRH, White City, London W12 0BZ, United Kingdom

[b] Shell Global Solutions International B.V., Energy Transition Campus Amsterdam, Grasweg 31, 1031 HW, Amsterdam, The Netherlands

[c] Department of Chemical Engineering, Imperial College London, South Kensington, London SW7 2AZ, United Kingdom

[d] Department of Earth Science and Engineering, Imperial College London, South Kensington, London SW7 2AZ, United Kingdom.

*** Corresponding author: anthony@imperial.ac.uk.**

Section S1. Voltammetry of MB

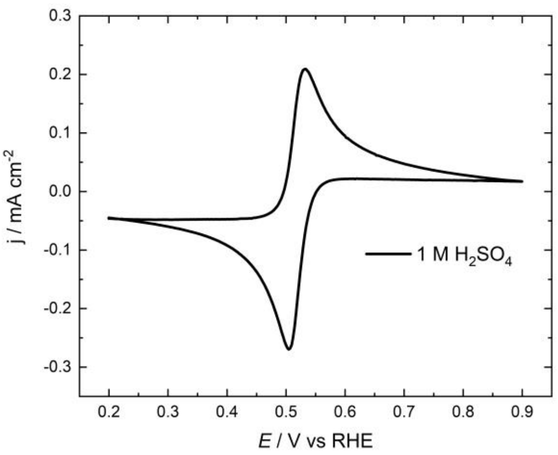

Figure S1: Cyclic voltammetry at a 50 mV s^{-1} scan rate for 1 mmol MB Ar-sat. 1 M H_2SO_4.

Section S2. Energy density of MB-H_2 RFC as function of hydrogen compression

Cell voltage of the H_2-MB rechargeable fuel cell:

$$E_{cell} = E^o_{cell} + \frac{RT}{2F} ln \frac{(p_{H_2}/p^o) a_{MB^{2+}}}{a_{R-MB} a_{H^+}{}^2} \quad \text{Equation S1}$$

Table S1: Theoretical volumetric energy density for the hydrogen gas and methylene blue electrolyte an of a H_2-MB rechargeable fuel cell at various H_2 pressures and 1.22 mol dm^{-3} (65.40 Ah dm^{-3}) MB.

P **(H_2) / Bar**	**1.00**	**13.67**	**100.00**
Q **(H_2) / Ah L^{-1}**	4.78	65.40	478.41
**E_{cell} / V**	0.520	0.554	0.579
**E_V / Wh dm^{-3}**	9.12	18.11	78.72

H_2-MB cell operating at STP, with H_2 pump after the outlet for compressed H_2 storage.

P **(H_2) / Bar**	**1.00**	**13.67**	**100.00**
Q **(H_2) / Ah L^{-1}**	4.78	65.40	478.41
**E_{cell} / V**	0.520	0.453	0.402
**E_V / Wh dm^{-3}**	9.12	14.81	54.65

Section S3. Definition and description of the "Theoretical power density"

By applying Equation S1, the limiting current was measured during rotating disk voltammetry, and the *D* coefficient calculated was found to be 1.51x10^{-6} cm^2 s^{-1} from the slope of i_L vs $\omega^{1/2}$, using the kinematic viscosity, v = 1.27x10^{-3} cm^2 s^{-1} for 1.0 M sulfuric acid and the slope of 0.0479 mA cm^{-2} $s^{1/2}$ $rad^{-1/2}$

$$i_L = 0.62nFAD^{2/3}v^{-1/6}\omega^{1/2}c* \qquad \text{Equation S1}$$

From intercept of Fig 1d, j_0 was determined to be 1.67 mA cm^{-2} (i_0 =3.28x10^{-4} A), and by applying Equation S2, k_0 = 8.65x10^{-3} cm s^{-1}.

$$i_k = nFAk_0c* \qquad \text{Equation S2}$$

The theoretical power density[2] of a positive electrolyte output defined by *iR*-free standard cell voltage vs the $H_2/2H^+$ negative cell reaction at an overpotential of RT/F (25.7 mV at 298K). As a point of reference for each mass transfer coefficient (k_m): for a 1 cm s^{-1} superficial flow rate of Fe^{2+}/Fe^{3+} electrolyte, a k_m of 1.40x10^{-3} is used, [1] and this is modified to the species of interest by noting that k_m is proportional to $D^{2/3}$.

$$\frac{1}{k_{comp}} = \frac{1}{k_0} + \frac{1}{k_m} \qquad \text{Equation S3}$$

The volumetric energy density E_V is for the fully charged species. [2]

$$3.6 * k_{comp}\ (\text{cm s}^{-1}) * E_v\ (\text{Wh L}^{-1}) = P_{\text{intrinsic}}\ (\text{W cm}^{-2}) \qquad \text{Equation S4}$$

Table S2: Physical and electrochemical parameters for aqueous positive H_2-*X* electrolyte redox couples.

	E **/ V**	***Electrolyte Solubility*** ***/ mol** dm^{-3}*	***E$_V$*** ***/ Wh L^{-1}***	***k$_o$*** ***/ x10^{-3} cm s^{-1}***	***D*** ***/ x10^{-6}cm^2 s^{-1}***	***$(D/D_{Fe(II/III)})^{2/3}$***	***k$_m$*** ***/x10^{-4} cm s^{-1}***	***k$_{comp}$*** ***/x10^{-4} cm s^{-1}***	***Ref.***	***Comments***
BB3	0.54	2.5 in 3.5 M H_2SO_4:AA<br>(1:1 by volume)	72.4	2.87<br>1.95 (GC only)	0.11	0.0649	0.911	0.828	[3]	*E* in 3.5 M H_2SO_4<br>Has not been tested in H_2-X RFC
BPTS	0.93	1.1 in 1 M H_2SO_4	54.2	0.112	1.84	0.425	5.96	0.943	[4]	Has not been tested in H_2-X RFC
BQDS	0.86	0.65 in 1 M H_2SO_4	182.2	0.155	3.80	0.689	9.66	1.34	[5, 6]	122 mW cm^{-2} peak power for H_2-BQDS
DHBS	0.71	0.8 in 1 M H_2SO_4	30.4	0.552	4.28	0.745	10.5	3.61	[7, 8]	Has not been tested in H_2-X RFC
DHDMBS	0.82	2 in 1 M H_2SO_4	87.9	0.13	4.12	0.727	10.2	1.15	[9]	Has not been tested in H_2-X RFC
Fe^{2+}/Fe^{3+}	0.77	0.7 [$Fe_2(SO_4)_3$]<br>0.8 03[$FeCl_3$]	28.9 [$Fe_2(SO_4)_3$]<br>16.5 [$FeCl_3$]	1.60	3.77<br>6.46	0.685<br>0.981	9.61<br>13.8	6.00<br>7.40	[1, 10, 11]	Both only 40 mL min^{-1} flow rate. Fe sulfate has higher viscosity.<br>147 and 207 mW cm^{-2} peak power respectively<br>k is assumed to be unaffected by anion present
VO^{2+}/VO_2^+	1.00	1.0	26.8	0.068	2.80	0.562	7.88	6.26	[12]	*k* & *D* in 1 M H_2SO_4
MB	0.52	1.2	33.4	8.65	1.51	0.373	5.23	4.93	*this work*	*k* & *D* in 1 M H_2SO_4<br>238 mW cm^{-2} peak power for H_2-MB

Section S4. High frequency resistance measurements of cell during charge/discharge

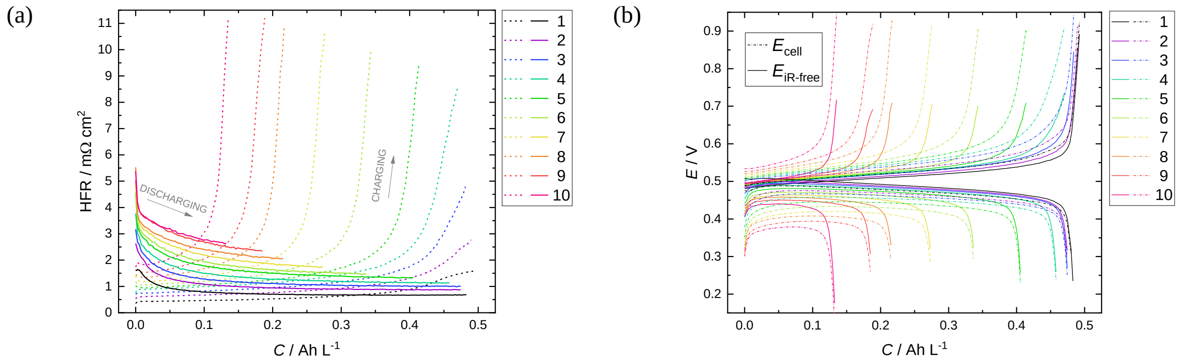

Figure S2: (a) High frequency resistance measured at 7.5 kHz during charging (dotted lines) and discharging (solid lines) of the 10 mM MB-H_2 *Nafion 212* IEM-containing RFC cell, and (b) the measured cell potential (E_{cell}) and the iR-compensated cell potential ($E_{iR\text{-}free}$) calculated from the HFR data.

Section S5. Determination of optical molar extinction coefficient of MB in water

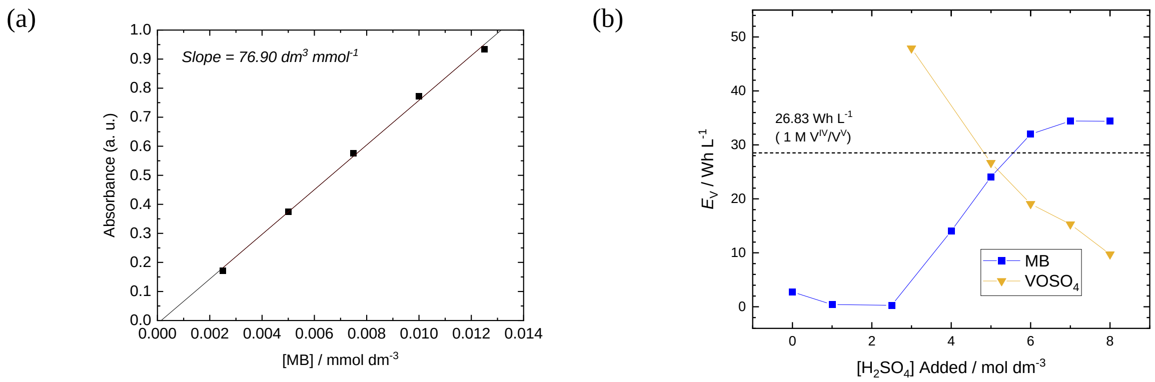

Figure S3: (a) Determination of the extinction coefficient of MB in water using the absorbance at 663 nm in 1 cm quartz cuvette, and (b) the concentration of MB and solubility of $VOSO_4$ versus the sulfuric acid concentration in the original solute ($VOSO_4$ solubility data from [13]).

Section S6. Concentration of MB in sulfuric acid of different concentrations

Sulfuric acid solutions and MB were mixed by stirring for 24 h. The samples were then filtered. Using a micropipette, a known volume of the filtrate was extracted. For the N212 cycling analysis a sample of the electrolyte was extracted. These were then each diluted (by volume) with deionised water until a sufficient dilution such that $0 < A_{663\ nm} < 0.9$ was achieved and the concentration could be analyzed by UV-Vis spectroscopy in a 3mL, 1 cm path length quartz glass cell (*Hellma*).

Table S3: Solubility calculation for MB-saturated H_2SO_4 solutions using the extinction coefficient at 663 nm of MB in water, calculated to be 76.90 mM cm^{-1}. Results are plotted above in Figure S3b.

[H_2SO_4] / M	*A_{663nm} (a.u.)*	*Dilution Factor ($x10^3$)*	*Concentration of MB* / mM
**0.0**	0.469	16	98
**1.0**	0.145	8	15
**2.5**	0.153	5	10
**4.0**	0.240	160	499
**5.0**	0.164	400	855
**6.0**	0.273	320	1137
**7.0**	0.294	320	1224
**8.0**	0.294	320	1223
N212 post-cycling analysis	0.565	1	7.34

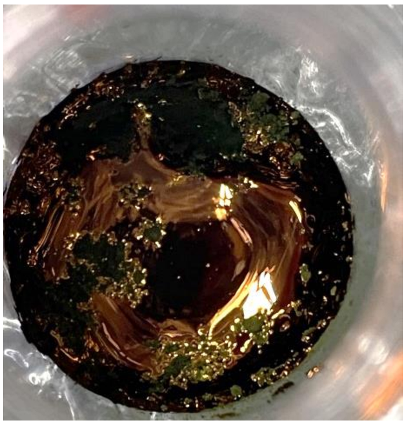

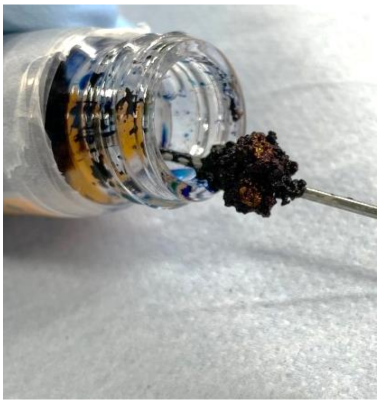

Figure S4: Photographs of MB displaying a gold-colour lustre after the addition of a highly concentrated sulfuric acid solution insufficient to fully solvate it.

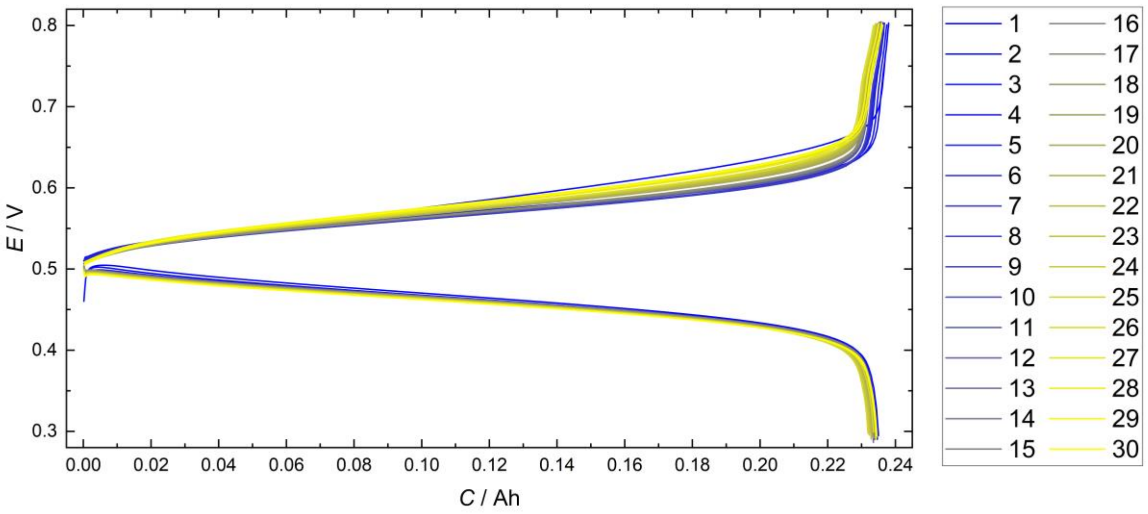

Figure S5: Voltage vs capacity profiles of a 0.1 M (50 mL) MB-H_2 RFC cell over 30 complete charge-discharge cycles at 50 mA cm^{-2} galvanostatic current.

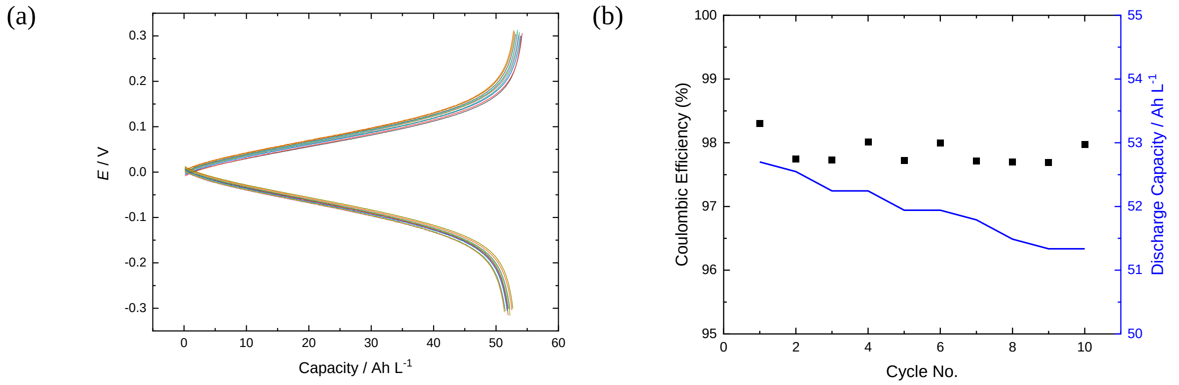

Figure S6: (a) Cell voltage vs time profiles and (b) figures of merit, for a 10-cycle test of 18 mL (vs 22 mL) 1.2 M concentration MB-MB symmetric cell using a DMBP-TB membrane with applied galvanostatic cycling current of 200 mA cm^{-2} and 0.3 V overpotential cut-off during charge and discharge.

References

(1) You, X.; Ye, Q.; Cheng, P. The Dependence of Mass Transfer Coefficient on the Electrolyte Velocity in Carbon Felt Electrodes: Determination and Validation. *J. Electrochem. Soc.* **2017**, *164* (11), E3386-E3394.
(2) Cannon, C. G.; Klusener, P. A. A.; Brandon, N. P.; Kucernak, A. Aqueous Redox Flow Batteries: Small Organic Molecules for the Positive Electrolyte Species. *ChemSusChem* **2023**, *16*, e202300303.
(3) Li, H.; Fan, H.; Ravivarma, M.; Hu, B.; Feng, Y.; Song, J. A stable organic dye catholyte for long-life aqueous flow batteries. *Chem. Commun.* **2020**, *56* (89), 13824-13827.
(4) Lantz, A. W.; Shavalier, S. A.; Schroeder, W.; Rasmussen, P. G. Evaluation of an Aqueous Biphenol- And Anthraquinone-Based Electrolyte Redox Flow Battery. *ACS Appl. Energy Mater.* **2019**, *2* (11), 7893-7902.
(5) Rubio-Garcia, J.; Kucernak, A.; Parra-Puerto, A.; Liu, R.; Chakrabarti, B. Hydrogen/functionalized benzoquinone for a high-performance regenerative fuel cell as a potential large-scale energy storage platform. *J. Mater. Chem. A* **2020**, *8* (7), 3933-3941.
(6) Yang, B.; Hoober-Burkhardt, L.; Krishnamoorthy, S.; Murali, A.; Prakash, G. K. S.; Narayanan, S. R. High-Performance Aqueous Organic Flow Battery with Quinone-Based Redox Couples at Both Electrodes. *J. Electrochem. Soc.* **2016**, *163* (7), A1442-A1449.
(7) Xu, Y.; Wen, Y.; Cheng, J.; Yanga, Y.; Xie, Z.; Cao, G. Novel organic redox flow batteries using soluble quinonoid compounds as positive materials. *WNWEC 2009 - 2009 World Non-Grid-Connected Wind Power and Energy Conference* **2009**, 475-478.
(8) Yang, B.; Hoober-Burkhardt, L.; Wang, F.; Surya Prakash, G. K.; Narayanan, S. R. An Inexpensive Aqueous Flow Battery for Large-Scale Electrical Energy Storage Based on Water-Soluble Organic Redox Couples. *J. Electrochem. Soc.* **2014**, *161* (9), A1371-A1380.
(9) Hoober-Burkhardt, L.; Krishnamoorthy, S.; Yang, B.; Murali, A.; Nirmalchandar, A.; Prakash, G. K. S.; Narayanan, S. R. A New Michael-Reaction-Resistant Benzoquinone for Aqueous Organic Redox Flow Batteries. *J. Electrochem. Soc.* **2017**, *164* (4), A600-A607.
(10) Tucker, M. C.; Srinivasan, V.; Ross, P. N.; Weber, A. Z. Performance and cycling of the iron-ion/hydrogen redox flow cell with various catholyte salts. *J. Appl. Electrochem.* **2013**, *43* (7), 637-644.
(11) Stephenson, D.; Kim, S.; Chen, F.; Thomsen, E.; Viswanathan, V.; Wang, W.; Sprenkle, V. Electrochemical Model of the Fe/V Redox Flow Battery. *J. Electrochem. Soc.* **2012**, *159* (12), A1993-A2000.
(12) Yufit, V.; Hale, B.; Matian, M.; Mazur, P.; Brandon, N. P. Development of a Regenerative Hydrogen-Vanadium Fuel Cell for Energy Storage Applications. *J. Electrochem. Soc.* **2013**, *160* (6), A856.
(13) Rahman, F.; Skyllas-Kazacos, M. Solubility of vanadyl sulfate in concentrated sulfuric acid solutions. *J. Power Sources* **1998**, *72* (2), 105-110.
